# Pylorus-Preserving Total Pancreatectomy for Intraductal Papillary Mucinous Neoplasm in the Setting of Previous Roux-en-Y Cystjejunostomy for Pancreatic Pseudocyst

**DOI:** 10.1089/pancan.2019.0015

**Published:** 2020-01-13

**Authors:** Christina M. Stuart, Megan P. Lundgren, Harish Lavu, Charles J. Yeo

**Affiliations:** ^1^ Sidney Kimmel Medical College at Thomas Jefferson University, Class of 2020, Philadelphia, Pennsylvania.; ^2^ Department of Surgery, Thomas Jefferson University Hospital, Philadelphia, Pennsylvania.; ^3^ Section of Hepatopancreatobiliary Surgery, Department of Surgery, Thomas Jefferson University Hospital, Philadelphia, Pennsylvania.; ^4^ Pancreas, Biliary and Related Cancer Center, Jefferson Health, Philadelphia, Pennsylvania.

**Keywords:** cystjejunostomy, intraductal papillary mucinous neoplasm, pylorus-preserving total pancreatectomy

## Abstract

**Background:** Intraductal papillary mucinous neoplasms (IPMNs) are cystic lesions of the pancreas with malignant potential. The Sendai and Fukuoka criteria offer guidelines for surgical management of an IPMN.

**Presentation:** A 69-year-old patient with a history of recurrent pancreatitis presented with steatorrhea and unintentional weight loss. Upon workup, he was found to have an IPMN, for which he met Sendai and Fukuoka criteria for surgical management. At the time of surgery, the patient's reported operative history was remarkable only for cholecystectomy; however, during the procedure, he was found to have a Roux-en-Y limb of jejunum attached to the head of the pancreas. Postoperative discussion with the patient and family revealed that this was likely the result of a past cystjejunostomy procedure used to drain what may have been a pancreatic pseudocyst that had developed after a bout of severe acute pancreatitis. Ultimately, the previously created Roux-en-Y limb was used in the reconstruction after specimen excision by total pancreatectomy.

**Conclusions:** Main duct IPMNs have a high incidence of carcinoma. Those that meet Sendai and Fukuoka criteria should be surgically managed. In this study we present a case of IPMN management by total pancreatectomy with unique reconstruction using a previously created Roux-en-Y limb.

## Introduction

Intraductal papillary mucinous neoplasms (IPMNs) are cystic lesions with the potential for progression to pancreatic cancer. They are anatomically classified as main duct (MD-IPMN), branch duct (BD-IPMN), or mixed-type based on the extent to which they involve the pancreatic ductal system. The patient discussed here was found to have an MD-IPMN, for which the risk of carcinoma *in situ* or invasive carcinoma is ∼70%.^1^

In 2004, the first international consensus guidelines for the management of IPMNs were put forth. Known as the Sendai criteria, these guidelines stated that lesions should be considered for surgical resection if they are >3 cm in size, have a solid component or mural nodules, or have a >5 mm dilated main pancreatic duct.^1^ Later in 2012, after several validation studies, the Fukuoka criteria built on these and tiered them out further into high risk and worrisome categories, specifically with modified ductal dilation criteria of >10 mm.^2^ Today, both are often used to decide whether a patient with IPMN should be managed with surgery, options for which include pancreaticoduodenectomy, distal pancreatectomy, segmental resection of the tumor, or total pancreatectomy.^3,4^

## Case Description

**FIG. 1.**
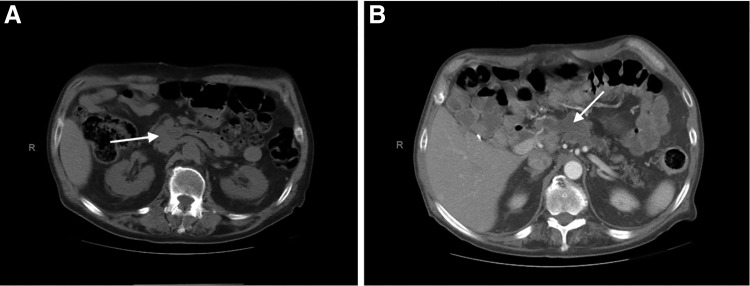
CT scans, with *arrows* showing cystic mass **(A)** and diffuse ductal dilation **(B)**.

The patient is a 69-year-old male with a medical history of diabetes, hypertension, and a remote history of several bouts of pancreatitis who presented to clinic with a 6-month history of steatorrhea and an unintentional 20 lb weight loss. During this visit, the patient reported an operative history of an open cholecystectomy by a right paramedian incision, and an appropriate scar was noted on physical examination. No formal records were available. Workup with CT imaging revealed a 2.1 × 7.0 cm multicystic lesion with mural nodularity in the body of the pancreas (Fig. 1A) and diffuse pancreatic ductal dilation throughout (Fig. 1B). Endoscopic ultrasound and the fine needle aspiration (FNA) measured the duct at 12.6 mm in the head, 14 mm in the neck, and 20 mm in the body, and after endoscopic tissue retrieval, the pathology returned as a mucinous neoplasm with focal atypia. A working diagnosis of IPMN was made. The patient's serum CA-19-9 was borderline elevated at 37 U/mL.

**FIG. 2.**
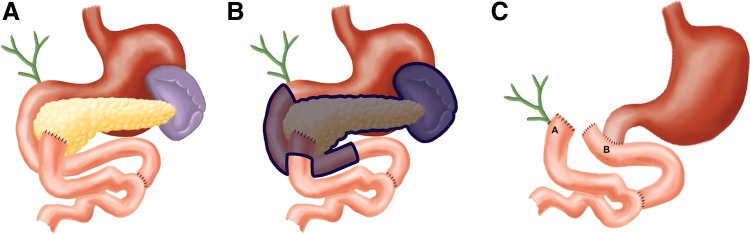
Preoperative anatomy **(A)**, highlighted surgical specimen **(B)**, and reconstructed anatomy **(C)** where A represents the newly created hepaticojejunostomy and B represents the new duodenojejunostomy.

The patient described met both Sendai and Fukuoka criteria and so was taken to the operating room for definitive treatment. In the operating room, the pancreas was found to be grossly abnormal with diffuse distortion of the duct throughout the head, body, and tail, thus warranting a pylorus-preserving total pancreatectomy and en-bloc splenectomy to safely remove all of the neoplastic tissue. During the procedure, a Roux-en-Y limb was discovered that had previously been brought up to the head of the pancreas in the antecolic position (Fig. 2 left). Intraoperative discussion among the surgical team concluded that this had presumably been performed to drain what may have been a pancreatic pseudocyst identified at the time of the patient's prior recurrent pancreatitis attacks. This was later confirmed upon postprocedural discussion of findings with the patient.

In the operating room, the decision was made to divide the Roux-en-Y limb preserving most of its length and its mesentery. The required pylorus-preserving total pancreatectomy was then safely performed. The cranial portion of the Roux-en-Y limb was used to perform the standard biliary–enteric reconstruction as an end-to-side hepaticojejunostomy, and alimentary reconstruction was completed by standard end-to-side duodenojejunostomy (Fig. 2 right) to the proximal most jejunum in an antecolic manner. The patient tolerated the procedure well.

Final surgical pathology revealed a 5.5 cm IPMN with low-grade dysplasia and surrounding chronic pancreatitis, as well as a 0.4 cm well-differentiated Ki-67 positive pancreatic endocrine tumor with Ki-67 proliferative index <1%. The patient's postoperative course was uneventful and he was discharged home on postoperative day 5. He was seen in the outpatient setting at a routine 1-month follow-up visit, at which time he was found to be in excellent condition and spirits, and overall, doing well.

## Discussion

Total pancreatectomy for the treatment of precancerous lesions such as IPMNs has been decreasingly recommended in favor of parenchyma-sparing resections.^5^ However, in select cases involving diffuse distortion of the duct, such as that described here, extensive resection or total pancreatectomy still represent the best choice for MD-IPMN^6^ as they are often accompanied by synchronous or metachronous multicentric lesions.^7,8^ In this study, total pancreatectomy was elected as parenchyma-sparing resection with negative margins would have resulted in an incredibly diminutive piece of normal pancreas. It has been suggested that in cases wherein the residual pancreas is <5 cm, anastomoses can be technically difficult with uncertain long-term benefits.^8^ In the case described here, it was clear that the required pancreatojejunostomy would have been both impractical and unsafe. However, if possible, we too recommend parenchyma-sparing resection with routine use of intraoperative frozen sectioning to achieve R0 resection.

Successive resections with iterative frozen sectioning can limit the risk of a wide range of complications. Extended resections such as total pancreatectomy are associated with high morbidity^9^ and candidates should be prepared for postoperative outcomes central to the loss of the exocrine and endocrine functions of the pancreas. The most common morbidities associated with exocrine insufficiency include weight loss and diarrhea, the latter of which has been shown to affect patients' quality of life.^7^ With regard to endocrine insufficiency, there are both short- and long-term serious sequelae and complications due to the development of “brittle” diabetes, characterized by wide variations or “swings” in blood glucose. At our institution, routine referral to endocrinology after total pancreatectomy has resulted in good glycemic control and self-management. Thus, we strongly recommend consultation with a specialist in pancreatogenic diabetes after total pancreatectomy, as was done with this patient.

The novelty of this case lies in the patient's history of recurrent acute pancreatitis and subsequent reported pseudocyst. Acute pancreatitis is an inflammatory process of the pancreas with pathophysiology related to enzymatic autodigestion.^10^ Local complications include pancreatic pseudocysts,^11^ which are encapsulated collections of fluid that arise due to disruption of the pancreatic ducts.^12^ Statistically, the majority of cysts appearing after pancreatitis are pseudocysts^13^; however, there are occasions wherein the discovered cyst is, in fact, a cystic tumor, especially when the patient has a modest history of smoking or alcohol consumption. Previous attacks of acute pancreatitis are not uncommon in IPMNs, in fact 12% of patients with IPMNs have a history of chronic pancreatitis and 2% of all chronic pancreatitis diagnoses are associated with IPMNs.^14^ Recurrent episodes of unexplained pancreatitis should, therefore, direct the attention to the possibility of their existence.

The inadvertent drainage of a pancreatic cystic tumor, misdiagnosed as a pseudocyst, has obvious consequences. It is, therefore, mandatory to ascertain the nature of the cyst in question before embarking on treatment. Initial diagnostic workup should begin with imaging. Radiological evidence of cystic tumors include thickened walls, internal septa, mural nodularity, papillary projections, solid components, and/or wall calcification.^15^ Cysts with these features should progress to surgical resection as outlined by the Sendai and Fukuoka criteria. Those without radiological evidence of neoplasia should proceed to FNA for cyst fluid analysis. If pseudocyst is confirmed, management depends on the patient's symptoms, characteristics, and location of the collection. Pseudocysts that fail to resolve with medical therapy can be managed through interventional techniques including percutaneous, endoscopic, and surgical methods.^16^ The preferred operative approach for most uncomplicated pseudocysts requiring surgical intervention is internal drainage for which there are three standard options: cystjejunostomy to a Roux-en-Y limb, cystgastrostomy, and cystdudodenostomy. Cystjejunostomy is the most versatile technique and has a slightly lower recurrence rate (7% vs. 10%) than cystgastrostomy.^17^ However, operative internal drainage of pancreatic pseudocysts has become less common as expertise in endoscopic techniques has increased.^16^

Here the patient had undergone a cystjejunostomy at an outside hospital in the 1980s as treatment for a reported pseudocyst. Interestingly, record of this procedure was not available to our operative team before surgery, neither disclosed by the patient nor through the medical record. In addition, the cystjejunostomy was not readily identified on preoperative imaging. Surgical history was clarified after postpancreatectomy discussion with the operative team in which it became clear that the patient and family had simply forgotten this procedure. Review of the literature has shown that patients recall only 41% of the visits noted in their medical records,^18^ and that almost half of the information that is remembered is incorrect.^19^ More specific to surgery, one in four patients do not accurately recall previous procedures.^20^ It is clear that the more remote in time the event, the more unreliable and unstable is its recall.^18^ Fortunately in the case described here, the discovery of the Roux-en-Y limb did not interfere with finalized functional reconstruction; however, it is clear that every effort should be made to avoid primary intraoperative recognition of altered anatomy.

## Conclusion

Pseudocysts are a common complication after a bout of pancreatitis; however, the possibility of pancreatic neoplasia must remain in the differential to preclude inappropriate drainage procedures. MD-IPMNs have a high incidence of carcinoma and those that meet Sendai and Fukuoka criteria should be surgically managed with parenchyma-sparing resection. However, total pancreatectomy may be necessary in extensive disease.

## Abbreviations Used

BD-IPMN: branch duct-intraductal papillary mucinous neoplasm
CT: computed tomography
FNA: fine needle aspiration
MD-IPMN: main duct-IPMN

## References

[1] Tanaka M, Chari S, Adsay V, et al. International consensus guidelines for management of intraductal papillary mucinous neoplasms and mucinous cystic neoplasms of the pancreas. Pancreatology. 2006;6:17–32 16327281 10.1159/000090023

[2] Tanaka M, Fernández-del Castillo C, Adsay V, et al. International consensus guidelines 2012 for the management of IPMN and MCN of the pancreas. Pancreatology. 2012;12:183–197 22687371 10.1016/j.pan.2012.04.004

[3] Bassi C, Procacci C, Zamboni G, et al. Intraductal papillary mucinous tumors of the pancreas. Verona University Pancreatic Team. Int J Pancreatol. 2000;27:181 10952400 10.1385/ijgc:27:3:181

[4] William Traverso L, Peralta EA, Ryan JA, et al. Intraductal neoplasms of the pancreas. Am J Surg. 1998;175:426–432 9600293 10.1016/s0002-9610(98)00039-7

[5] Arnelo U, Siiki A, Swahn F, et al. Single-operator pancreatoscopy is helpful in the evaluation of suspected intraductal papillary mucinous neoplasms (IPMN). Pancreatology. 2014;14:510–514 25287157 10.1016/j.pan.2014.08.007

[6] Del Chiaro M, Rangelova E, Segersvärd R, et al. Are there still indications for total pancreatectomy? Updates Surg. 2016;68:257–263 27605208 10.1007/s13304-016-0388-6 PMC5123621

[7] Watanabe Y, Ohtsuka T, Matsunaga T, et al. Long-term outcomes after total pancreatectomy: special reference to survivors' living conditions and quality of life. World J Surg. 2015;39:1231–1239 25582768 10.1007/s00268-015-2948-1

[8] Sauvanet A, Couvelard A, Belghiti J Role of frozen section assessment for intraductal papillary and mucinous tumor of the pancreas. World J Gastrointest Surg. 2010;2:352–358 21160843 10.4240/wjgs.v2.i10.352 PMC2999199

[9] Buscail E, Cauvin T, Fernandez B, et al. Intraductal papillary mucinous neoplasms of the pancreas and european guidelines: importance of the surgery type in the decision-making process. BMC Surg. 2019;19:115–110 31438917 10.1186/s12893-019-0580-y PMC6704670

[10] Felderbauer P, Müller C, Bulut K, et al. Pathophysiology and treatment of acute pancreatitis: new therapeutic targets—a ray of hope? Basic Clin Pharmacol Toxicol. 2005;97:342–350 16364048 10.1111/j.1742-7843.2005.pto_274.x

[11] Banks PA, Bollen TL, Dervenis C, et al. Classification of acute pancreatitis—2012: revision of the Atlanta classification and definitions by international consensus. Gut. 2013;62:102–111 23100216 10.1136/gutjnl-2012-302779

[12] Kozarek R Endotherapy for organized pancreatic necrosis: perspective on skunk-poking. Gastroenterology. 1996;111:820–823 8780591 10.1053/gast.1996.v111.agast961110820

[13] Warshaw AL, Christison-Lagay ER Pancreatic cystoenterostomy. In: Mastery of Surgery. Fischer JE, Bland KI (eds.) Lippincott Williams & Wilkins: New York, NY; pp. 1278–1288; 2007

[14] Talamini G, Zamboni G, Salvia R, et al. Intraductal papillary mucinous neoplasms and chronic pancreatitis. Pancreatology. 2006;6:626–634 17135772 10.1159/000097605

[15] Kim YC, Choi J, Chung YE, et al. Comparison of MRI and endoscopic ultrasound in the characterization of pancreatic cystic lesions. Am J Roentgenol. 2010;195:947–952 20858823 10.2214/AJR.09.3985

[16] Marino KA, Hendrick LE, Behrman SW, Surgical management of complicated pancreatic pseudocysts after acute pancreatitis. Am J Surg. 2016;211:109–114 26507289 10.1016/j.amjsurg.2015.07.020

[17] Newell KA, Liu T, Aranha GV, et al. Are cystgastrostomy and cystjejunostomy equivalent operations for pancreatic pseudocysts? Surgery. 1990;108:635 2218873

[18] Barsky AJ Forgetting, fabricating, and telescoping: the instability of the medical history. Arch Intern Med. 2002;162:981–984 11996606 10.1001/archinte.162.9.981

[19] Kessels RPC Patients' memory for medical information. J R Soc Med. 2003;96:219–222 12724430 10.1258/jrsm.96.5.219 PMC539473

[20] Hess LM, Method MW, Stehman FB, et al. Patient recall of health care events and time to diagnose a suspected ovarian cancer. Clin Ovarian Other Gynecol Cancer. 2012;5:17–23

